# Supplementary material for: BRCA2 associates with MCM10 to suppress PRIMPOL-mediated repriming and single-stranded gap formation after DNA damage
Source: Nat Commun. 2021 Oct 13;12:5966. doi: 10.1038/s41467-021-26227-6 (PMC8514439; doi:10.1038/s41467-021-26227-6)
Supplement: Supplementary file 1 — Supplementary information [file 41467_2021_26227_MOESM1_ESM.pdf]

Kang et al., Supplementary Figure 1

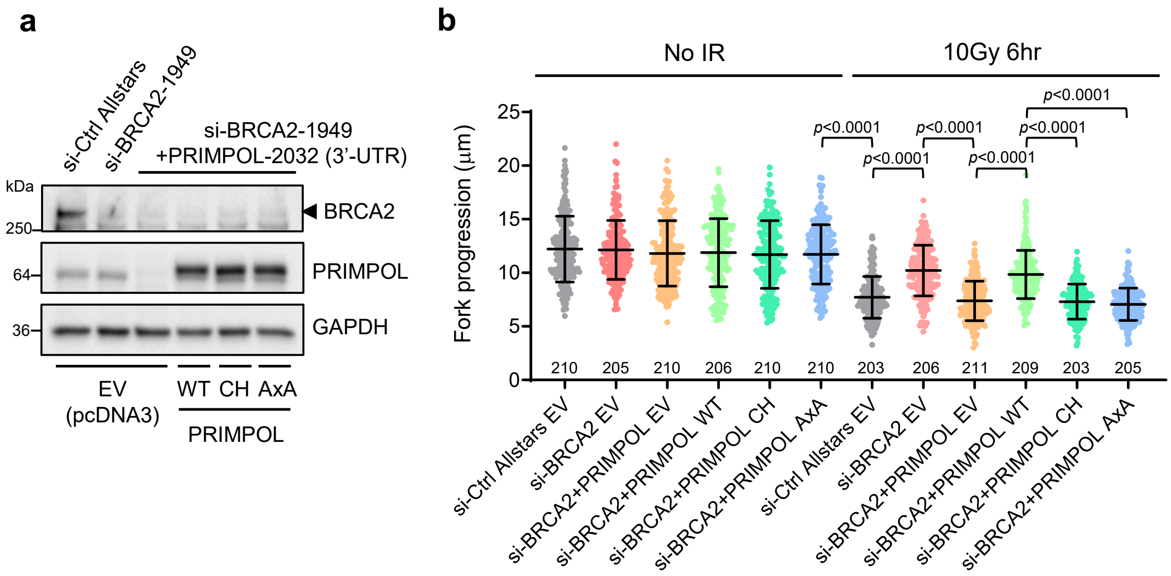

Supplementary Figure 1. PRIMPOL's enzymatic activities underlie the underrestrained replication fork progression in BRCA2-depleted cells after ionizing radiation (IR). **a** Representative western blot images showing the depletion of BRCA2 and PRIMPOL as well as transient expression of wild type (WT) and mutant PRIMPOL proteins in BRCA2 and PRIMPOL co-depleted cells. CH is a primase-dead, polymerase-competent PRIMPOL mutant, and AxA is a catalytic mutant. Similar results were obtained in n=2 independent experiments. **b** Replication tract lengths in BRCA2/PRIMPOL co-depleted cells expressing exogenous WT and mutant PRIMPOL before and after IR. Data are presented as means ± s.d., with the number of dots shown below each column. Similar results were obtained in n=2 independent experiments. P values are calculated using two-tailed unpaired Student' *t* test.

Kang et al., Supplementary Figure 2

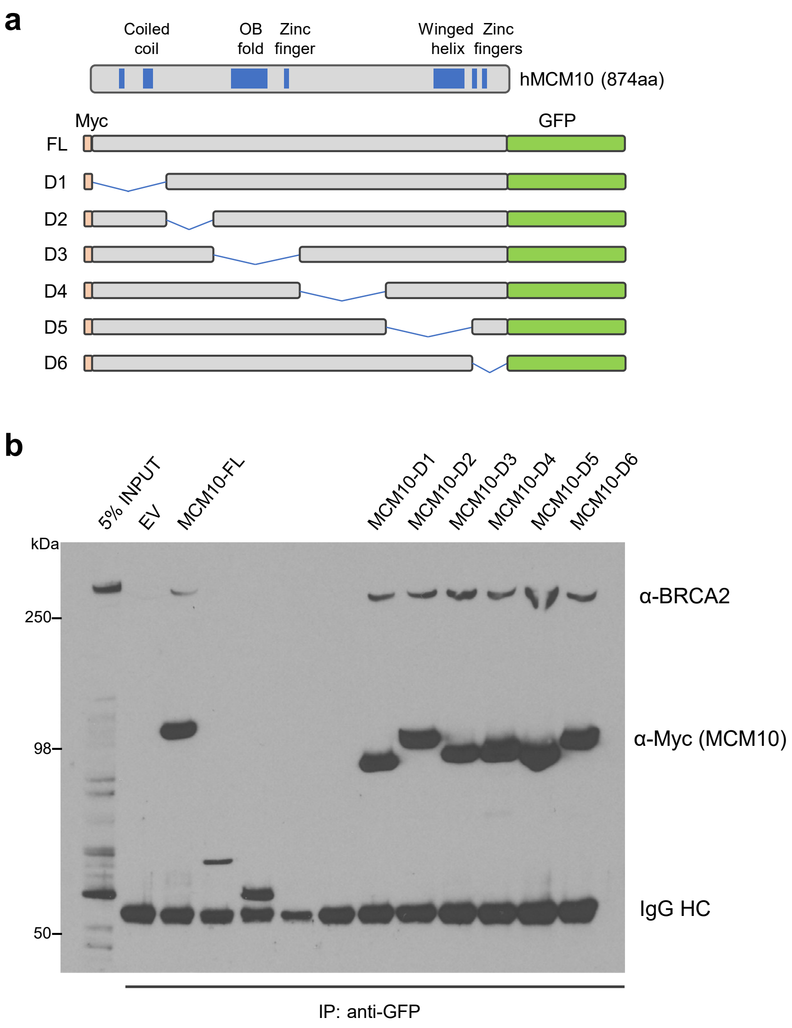

Supplementary Figure 2. Interaction between different MCM10 deletion mutants and BRCA2. **a** Schematic diagram of MCM10 domain structure and the 6 different deletions generated in a Myc-MCM10-GFP expression vector. **b** Co-IP of the overexpressed MCM10 deletion mutants with the endogenous BRCA2 in 293T cells. Similar results were obtained in 2 independent experiments.

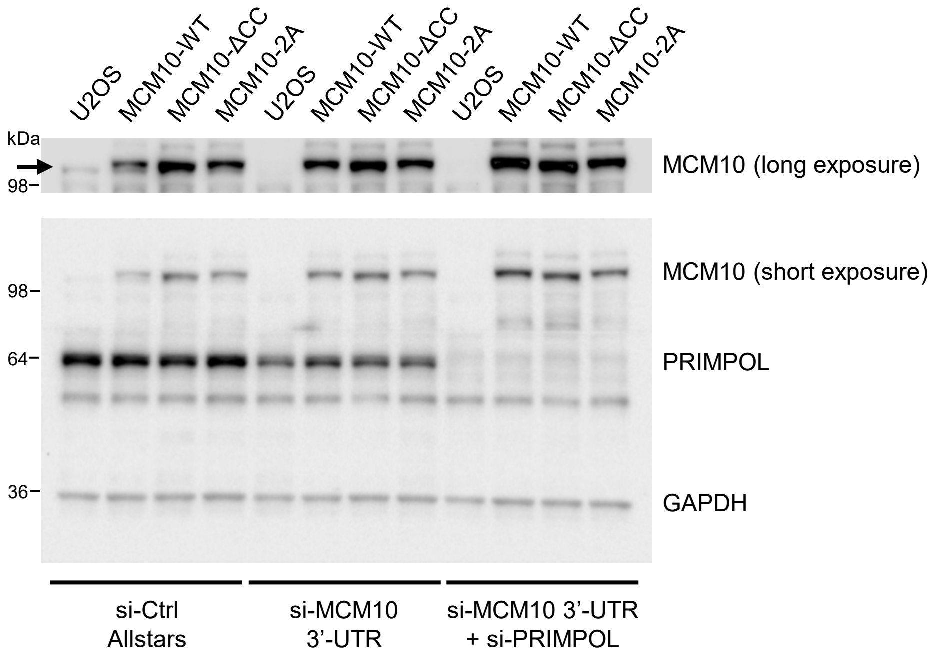

Supplementary Figure 3. siRNA mediated depletion of endogenous MCM10 alone or in combination with PRIMPOL in blank U2OS cells or U2OS cells stably expressing MCM10-WT, MCM10-ΔCC or MCM10-2A mutants. For MCM10, a pool of two siRNAs targeting the 3'-UTR of its mRNA were used. Black arrow indicates endogenous MCM10. Similar results were obtained in 3 independent experiments.

Kang et al., Supplementary Figure 4

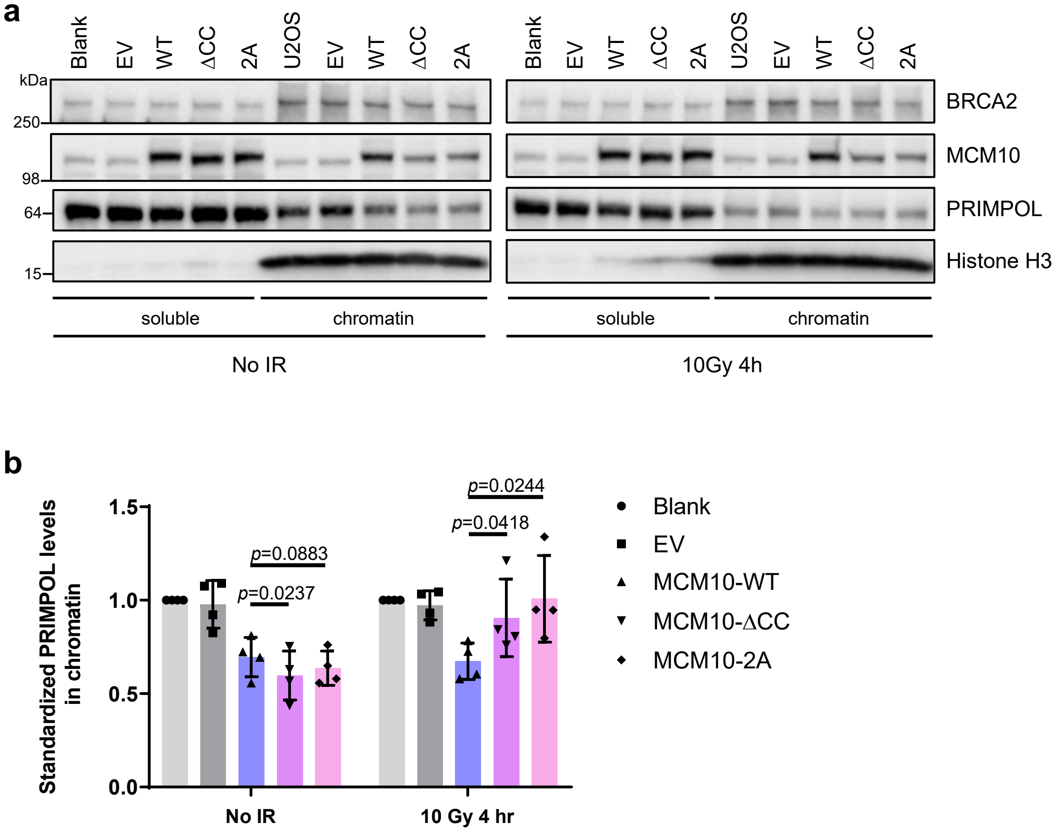

Supplementary Figure 4. Evidence that disruption of MCM10-BRCA2 interaction leads to increased PRIMPOL chromatin loading. **a** Western blots showing the distribution of PRIMOL in the soluble vs chromatin fractions in blank U2OS cells, empty vector (EV) expressing cells, and cells selectively expressing wild type (wt) and coiled-coil (CC) mutant MCM10 proteins before and after IR. In the 3 cell lines stably expressing WT and the mutant MCM10 proteins, endogenous MCM10 was depleted with a pool of 2 siRNAs targeting the 3'-UTR of its mRNA. Similar results were obtained in n=4 independent experiments. **b** Quantification of the amounts the PRIMPOL in the soluble and chromatin fractions before and after IR as in **a**. Data are presented as mean ± s.d. from n=4 independent experiments. P values are calculated using two-tailed paired Student's *t* test.

Kang et al., Supplementary Figure 5

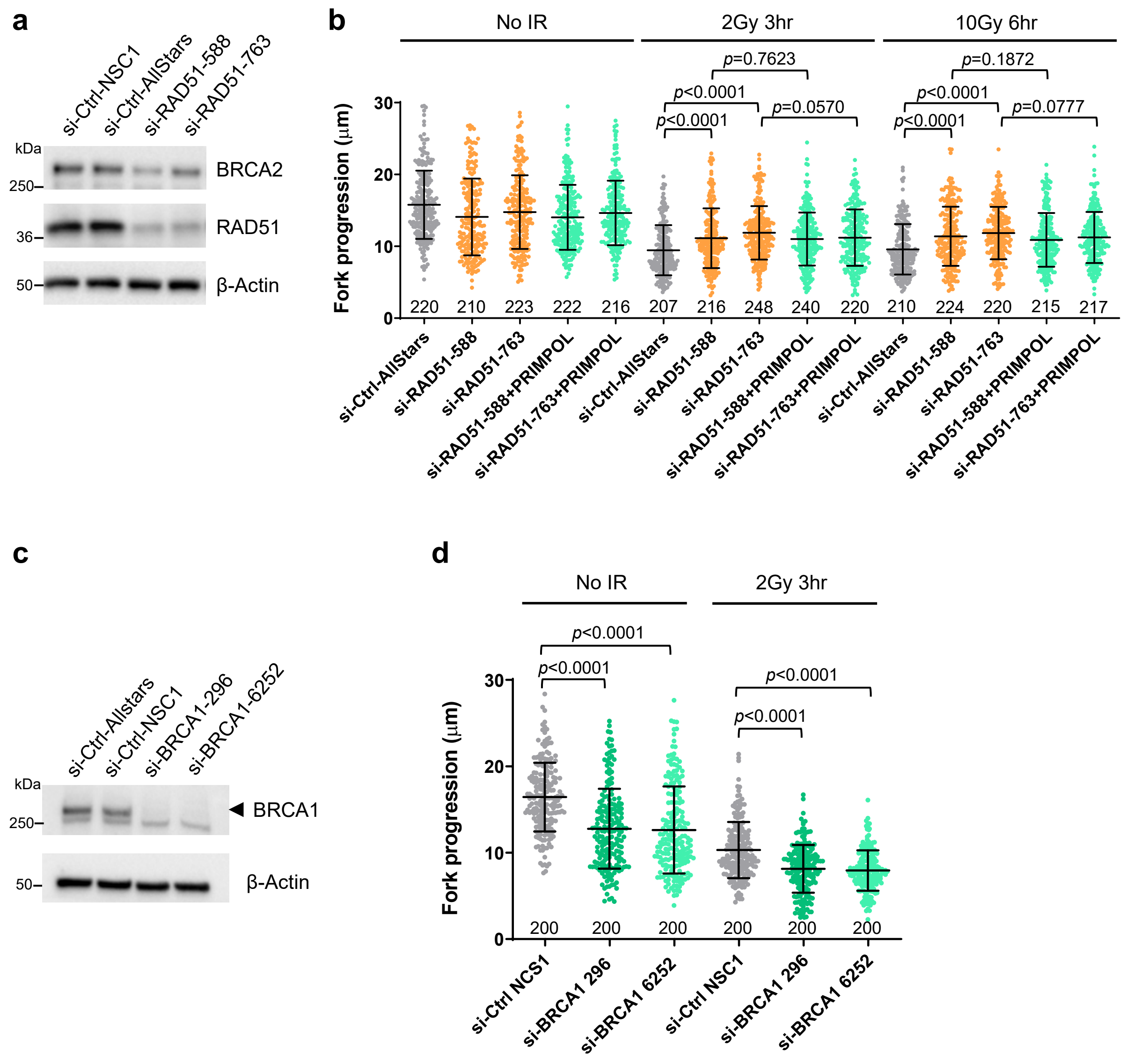

Supplementary Figure 5. Effect of RAD51 and BRCA1 depletion on replication fork progression after irradiation (IR). **a** Western blots showing depletion of RAD51 in U2OS cells with two different siRNAs. Note that depletion of RAD51 led to slowdown of cell growth and a moderate reduction of BRCA2 amount. Similar results were obtained in n=3 independent experiments. **b** Replication tract lengths in cells treated with control, RAD51, and RAD51+PRIMPOL siRNAs after IR. Data are presented as mean ± s.d., with the number of dots provided below each column. Similar results were obtained in n=2 independent experiments. **c** Western blots showing depletion of BRCA1 in U2OS cells with two different siRNAs. Similar results were obtained in n=3 independent experiments. **d** Replication tract lengths in BRCA1-depleted cells before and after IR. Data are presented as mean ± s.d., with the number of dots provided below each column. Similar results were obtained in n=3 independent experiments. For both **b** and **d**, p values are calculated using two-tailed unpaired Student's *t* test.

Kang et al., Supplementary Figure 6

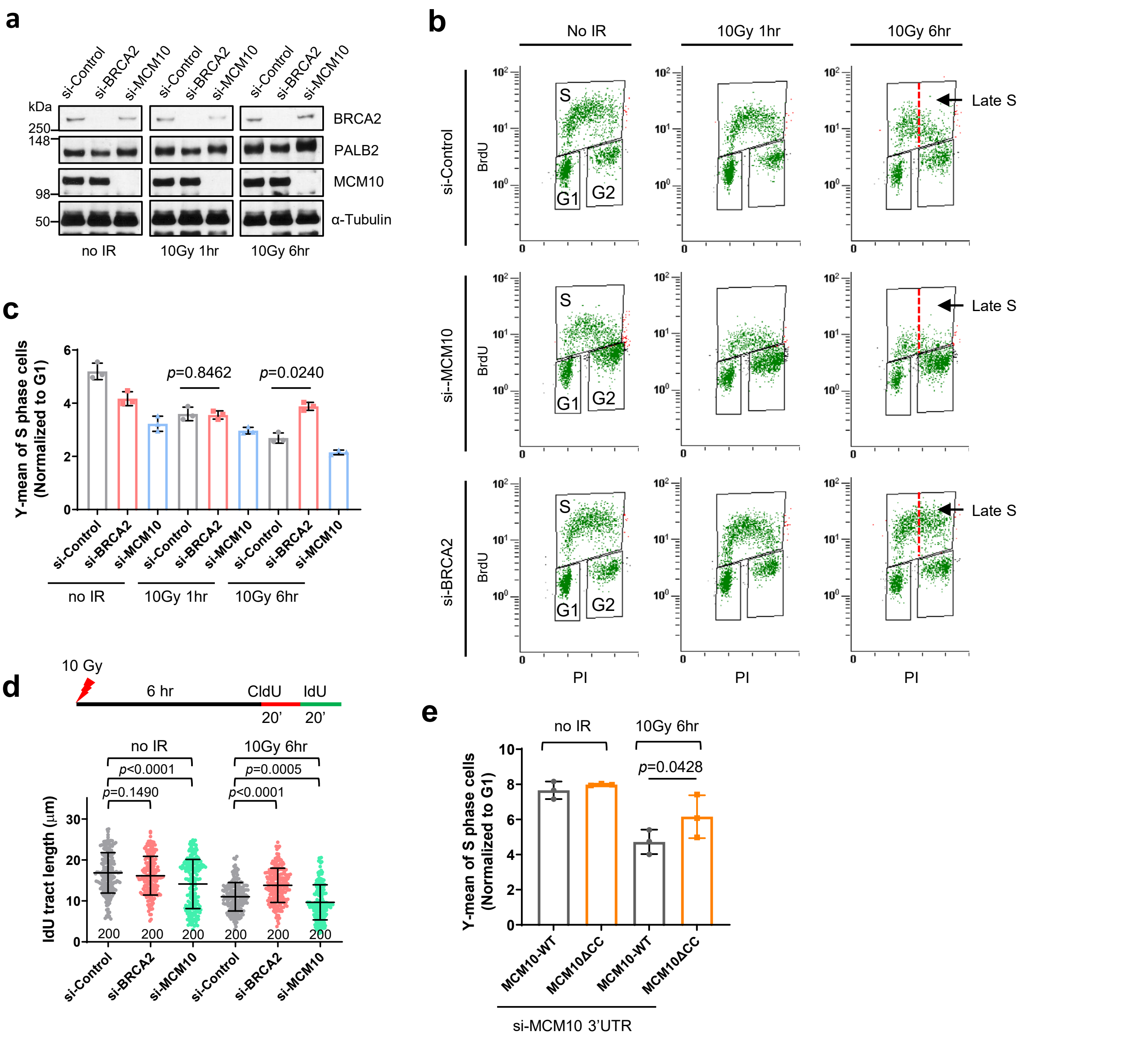

Supplementary Figure 6. Effect of loss of BRCA2, MCM10, or the MCM10-BRCA2 complex formation on DNA replication after irradiation (IR). **a** Representative western blots showing siRNA-mediated depletion of MCM10 and BRCA2 in U2OS cells. Similar results were obtained in n=3 independent experiments. **b** Representative plots showing BrdU incorporation in U2OS cells depleted of BRCA2 and MCM10, before and after IR. Similar results were obtained in 5 independent experiments. Gating strategy is provided in **Supplementary Figure 8.** **c** Quantified BrdU incorporation in **b**. For each condition, the mean value of S phase population was normalized against that of G1 population. Data are presented as mean ± s.d. from n=3 independent experiments. P values are calculated using two-tailed paired Student's *t* test. **d** Replication tract lengths in U2OS cells depleted of BRCA2 or MCM10 before and after IR. Data are presented as mean ± s.d. with the number of dots shown below each column. P values are calculated using two-tailed unpaired Student's *t* test. Similar results were obtained in 3 independent experiments. **e** Quantified BrdU incorporation in U2OS cells selectively expressing MCM10-WT and MCM10-ΔCC before and after IR. Data are presented as mean ± s.d. from 3 independent experiments ($n = 3$). P values are calculated using two-tailed paired Student's *t* test.

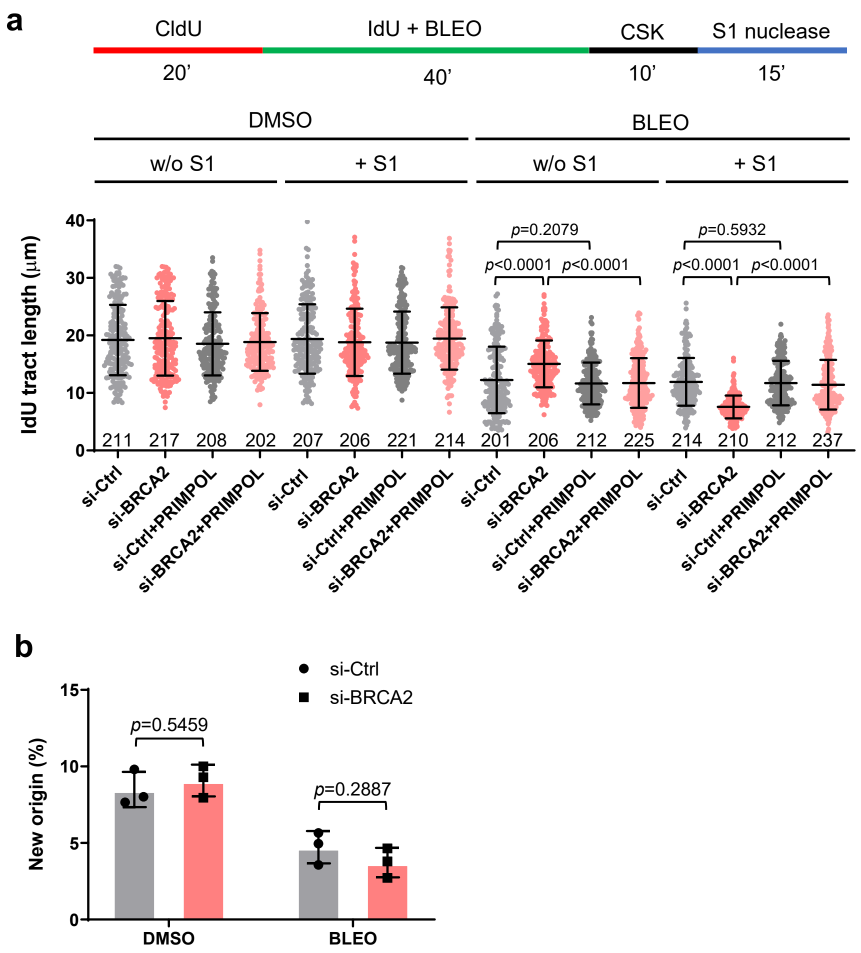

Supplementary Figure 7. DNA replication kinetics in U2OS cells depleted of BRCA2 either alone or in combination with PRIMPOL during bleomycin (BLEO) treatment. **a** Replication tract lengths and ssDNA gap formation in the cells before and during 1 μM BLEO treatment. Labeling and S1 nuclease assay schemes are shown on top. Replication speed for ongoing forks was scored by measuring the lengths of IdU tracts in CIdU/IdU positive tracts. Data are presented as mean ± s.d., with the number of dots shown below each column. P values are calculated using two-tailed unpaired Student’s *t* test. **b** New origin firing before and during BLEO treatment. New origin firing was scored by counting the percentage of IdU-only tracts. Data are presented as mean ± s.d. from n=3 independent experiments. P values are calculated using two-tailed unpaired Student’s *t* test.

Kang et al., Supplementary Figure 8

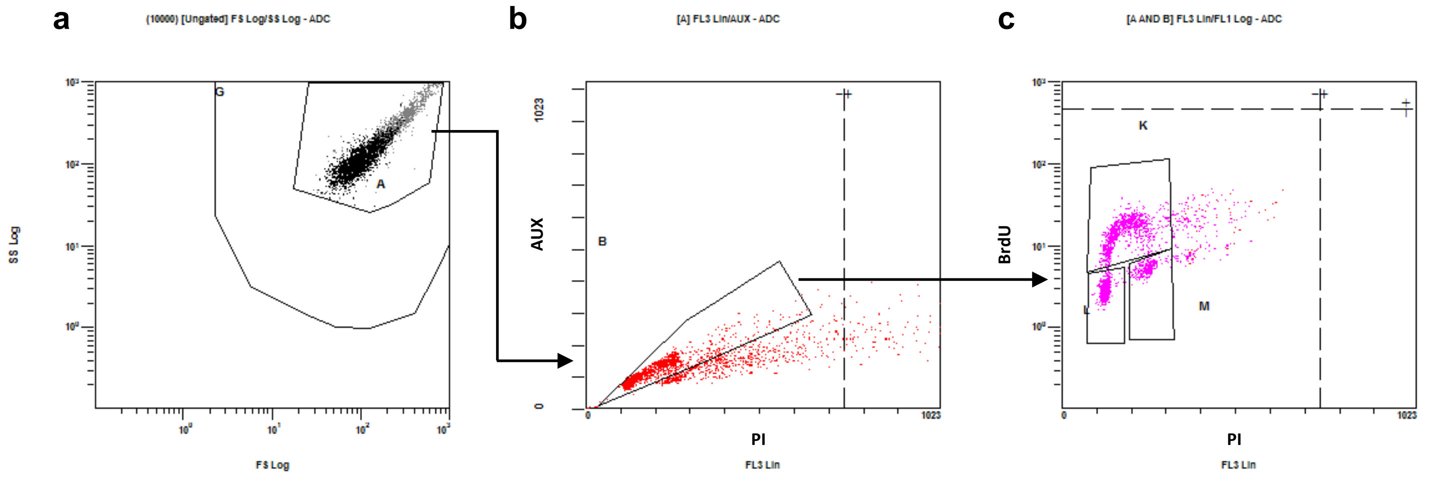

Supplementary Figure 8. Gating strategy used to determine cell cycle distribution and BrdU incorporation in cells. **a** Cells were first identified based on forward scatter (FS) and size scatter (SS). **b** Singlet cells were then selected based on propidium iodide (PI) content (FL3 Lin) and auxiliary parameter (AUX). **c** Singlet cells were separated into G1, S and G2/M phase cells based on PI (FL3 Lin) and BrdU (FL1) content.

Supplementary methods

Virus packaging and stable cell line generation

pOZ-FH-C-PALB2 or MCM10 retroviruses were packaged in 293T cells as follows. Cells were seeded into 6-well plates at 600,000 cells per well in 2 ml of medium and allowed to adapt overnight. Cells were transfected with a mixture of pOZ-FH-C-PALB2 or MCM10, gag-pol, and env plasmids (1 µg each) with 6 µl of X-tremeGENE HP (Roche, 6366236001). Approximately 30 hr after transfection, another 2 ml of medium was added to each well. Forty-eight hr after transfection, culture media were collected and spun at 2,000xg for 10 min, and the supernatants were used for viral transduction.

The day before virus collection, HeLa S3 or U2OS cells were seeded into 10 cm plates at $2x10^6$ cells per plate. On the following day, 2 ml of the above viral supernatant was added to each plate, followed by the addition of 12 µl of 8 mg/ml polybrene. Cells were grown for 48 hr and then transferred to 15 cm plates. Another 48-72 hr later, cells were harvested and resuspended in 8 ml of culture medium in 15 ml tubes. To select positive cells, 4 µl of Dynabeads Goat Anti-Mouse IgG (Invitrogen, 11033) coupled with anti-IL2Rα antibody (clone 7G7/B6, Millipore 05-170) were added, and the mixture were rocked at room temperature (RT) for 1 hr. Afterwards, the tubes were placed on a magnetic stand for 3-5 min. Media was then removed, cells attached to tube wall were gently resuspended in 8 ml of medium, and the tubes were put back on the magnetic stand for 3-5 min. Following media removal, selected cells were resuspended in 5 ml of fresh media, transferred to T25 flasks and grown to confluency. Cells were then expanded into 10 cm plates, grown to confluency and then selected for another round.

Tandem affinity purification of PALB2 complexes

Approximately 2×10^8 HeLa S3 cells stably expressing the FLAG-HA-double-tagged PALB2 were extracted with 10 ml of NETNG250 (20 mM Tris-HCl [pH 7.4], 250 mM NaCl, 10 mM NaF, 0.5% NP-40, 1 mM EDTA, 5% glycerol) with the cOmplete protease inhibitor cocktail (Roche, 11697498001). FLAG-HA–tagged PALB2 protein complexes were first isolated from the above extract using 50 µl of anti-FLAG M2 agarose beads (Sigma, A2220), eluted with 200 µl of 0.2 mg/ml FLAG peptide, and then further purified with 20 µl of anti-HA agarose beads (Sigma, A2095). The final material bound to the anti-HA beads was eluted with 50 µl of 0.1 M glycine (pH 2.5) and then neutralized with 0.1 volume of 1 M Tris-HCl (pH 8.5). Detailed protocol will be provided upon request.

Mitotic spread / chromosomal abnormality analysis

U2OS cells were seeded into 6-well plates at 150,000 cells per well and treated with siRNAs the day after. Cells were harvested 48 hr after siRNA transfection, spun down, and resuspended in 3 ml of hypotonic solution (0.075 M KCl). Cells were incubated at 37°C for 15 min and then 40 µl of fixative solution (3:1 Methanol:Acetic Acid) was added. Cells were spun down, resuspended in 1 ml of fixative solution, and incubated on ice for 45 min. Then, about 20 µl of the fixed cells were dropped onto the glass slides from a height of ~5 cm to obtain mitotic spreads. Slides were placed in 60°C warmer for 1 hr. After cooling down, slides were stained using Giemsa solution for 5 min, washed in distilled water, and air-dried. Slides were then observed and image taken under a microscope.

Reversal of PRIMOL siRNA effect by cDNA transfection (Supplementary Figure 2)

U2OS cells were first treated with siRNAs in 6-well plates for 36 hr. Culture media were then replaced, and cells were allowed to adapt in fresh media for 6 hr. Cells were then transfected with either empty vector (pcDNA3) or PRIMPOL constructs using X-tremeGENE HP (Roche). The amount of cDNA and reagents used were 0.25 µg and 1 µl, respectively. 36 hr after cDNA transfection, cells were collected for western blotting or labeled with CldU and IdU for DNA fiber analysis.

Cell fractionation (Supplementary Figure 4)

U2OS cells stably expressing different MCM10 proteins were trypsinized, washed with PBS, and resuspended in 100 µl ice cold NETNG-100 (20 mM Tris-HCl [pH7.4], 100 mM NaCl, 1 mM EDTA, 10 mM NaF, 0.5% NP-40 and 5% Glycerol). Cells were incubated on ice for 10 min with intermittent mixing by flicking. The mixtures were centrifuged at 2,000xg for 10 min, and the supernatants were collected as the soluble fractions. The pellets, or chromatin fractions, were resuspended in 100 µl NETNG-350 and sonicated in a Fisher Model 100 Sonic Dismembrator at setting 2 for 5 rounds or a total of 10 sec. Equal volumes of each fraction were loaded for western blotting analyses.

BrdU incorporation (Supplementary Figure 6)

U2OS cells were seeded into 6 well plates at 150,000 cells per well and treated with siRNAs for 48 hr and then with IR, where applicable. Cells were labeled with 20 µM BrdU for 20 min, trypsinized and transferred to Eppendorf tubes. Cells were spun down at 1500xg for 6 min, washed with 1%BSA/PBS, spun down, resuspended in 0.5 ml of 70% ethanol, and stored at -20°C overnight. To denature the DNA, cells were spun down, resuspended in 0.5 ml of 2N HCl with 0.5% Trition X-100 and incubated at room temperature for 30 min. To neutralize the acid, cells were spun down and resuspended in 0.5 ml of 0.1 M $Na_2B_4O_7$ (pH8.5). Cells were then spun down, loosened by flicking, and resuspended in 300 µl of 0.5% Tween 20/1% BSA/PBS. Twenty µl FITC anti-BrdU (BD Pharmingen, Cat. 347583) was then added, and the mixture was incubated on a rocker at 4°C overnight. Cells were then spun down and washed with Tween/BSA/PBS. Finally, cells were spun down, resuspended in PBS containing 1 µg/mL propidium iodide (PI), and analyzed by flow cytometry on a Beckman Coulter FC500 cytometer. Gating strategy is provided in Supplementary Figure 8.

Supplementary Table 1.

Primers used for MCM10, cloning mutagenesis and sequencing. Note that the 4A mutant was generated on top of the 2A mutant.

MCM10 Fwd	CGCGTCGACCCAACCATGGATGAGGAGGAAGACAATC
MCM10 Rev	GCGTGCGGCCGCTTATTTAAGGCTGTTCAGAAATTTAG
MCM10-D1 Fwd	GAAGAGGATCTGGTCGACTCTCCCCGGCCACCTC
MCM10-D1 Rev	GAGGTGGCCGGGGAGAGTCGACCAGATCCTCTTC
MCM10-D2 Fwd	CGTCTGCAAAAATCCCCTGTAGAGAAGACGACTCAACCCATCTGTGTGGAAGCC
MCM10-D2 Rev	GGCTTCCACACAGATGGGTTGAGTCGTCTTCTCTACAGGGGATTTTTGCAGACG
MCM10-D3 Fwd	GGAAGTTCTGGGGAAGCGGATCTGCAGTCCACC
MCM10-D3 Rev	GGTGGACTGCAGATCCGCTTCCCCAGAACTTCC
MCM10-D4 Fwd	GCTCAGCGCAAAGCGTCCTCCACGGACAGGATCC
MCM10-D4 Rev	GGATCCTGTCCGTGGAGGACGCTTTGCGCTGAGC
MCM10-D5 Fwd	CAGCCCCCTGCTCAGAAGTGCCGTGTCGTG
MCM10-D5 Rev	CACGACACGGCACTTCTGAGCAGGGGGCTG
MCM10-D6 Fwd	GAGAAACATCAGAGAAGTGGCGGCCGCAGGATCC
MCM10-D6 Rev	GGATCCTGCGGCCGCCACTTCTCTGATGTTTCTC
MCM10-ΔCC Fwd	CCTGCCCCCAGGCGAAAAGTAACAACAATTAAACAG
MCM10-ΔCC Rev	CTGTTTAATTGTTGTTACTTTTCGCCTGGGGGCAGG
MCM10-2A Fwd	GAGAAAACGAATGAAGAGGCGCAAGAGGAAGCAAGGAATTTGCAAGAGC
MCM10-2A Rev	GCTCTTGCAAATTCCTTGCTTCCTCTTGCGCCTCTTCATTCGTTTTCTC
MCM10-2D Fwd	GAGAAAACGAATGAAGAGGATCAAGAGGAAGATAGGAATTTGCAAGAGC
MCM10-2D Rev	GCTCTTGCAAATTCCTATCTTCCTCTTGATCCTCTTCATTCGTTTTCTC
MCM10-4A-2 Fwd	GGAATTTGCAAGAGCAAGCGAAGGCCGCACAAGAGCAGCTAAAAG
MCM10-4A-2 Rev	CTTTTAGCTGCTCTTGTGCGGCCTTCGCTTGCTCTTGCAAATTCC
SEQ-pOZ	CATCGCAGCTTGGATACA

Supplementary Table 2.

Sense strand sequences of siRNAs used in this study.

NSC1	UUCGAACGUGUCACGUCAAdTdT	
BRCA2-1949	GAAGAAUGCAGGUUUAAUAdTdT	
BRCA2-9025	GGCAAAUGUUGAAUGAUAAdTdT	
BRCA2-11170	CCUUAAGUCAGCAUGAUUAdTdT	
MCM10-3006	GAAGGUAAACAGAGAGCUAdTdT	(3′-UTR)
MCM10-3089	ACGAACACCCAGAGGCAAAdTdT	(3′-UTR)
HLTF-1208	GGAAUAUAAUGUUAACGAUdTdT	
HLTF-2841	GGAUUUGUGUUUACUCGUUdTdT	
SMARCAL1-1978	GAAUCUCACUUCCUCAAAAdTdT	
SMARCAL1-2743	GAAUAUAUCUUGGACCUACdTdT	
ZRANB3-1614	UGACAGUUCUGAAGAGUUAdTdT	
ZRANB3-3323	GAUCAGACAUCACACGAUUdTdT	
PRIMPOL-1138	AGAAAAGGCUACAGAGGAAdTdT	
PRIMPOL-1158	GCUGGACATCGAAUUCAAAdTdT	
PRIMPOL-2032	GCCUGAUGUCUGUGAGAUUdTdT	(3′-UTR)
BRCA1-296	GGAACCUGUCUCCACAAAGdTdT	
BRCA1-6252	GGAUCGAUUAUGUGACUUAdTdT	
RAD51-588	UCAGAGAUCAUACAGAUUAdTdT	
RAD51-763	GAAAGGCCAUGUACAUUGAdTdT	
